# Correction: Gender differences in predictors of intensive care units admission among COVID-19 patients: The results of the SARS-RAS study of the Italian Society of Hypertension

**DOI:** 10.1371/journal.pone.0257181

**Published:** 2021-09-02

**Authors:** Guido Iaccarino, Guido Grassi, Claudio Borghi, Stefano Carugo, Francesco Fallo, Claudio Ferri, Cristina Giannattasio, Davide Grassi, Claudio Letizia, Costantino Mancusi, Pietro Minuz, Stefano Perlini, Giacomo Pucci, Damiano Rizzoni, Massimo Salvetti, Riccardo Sarzani, Leonardo Sechi, Franco Veglio, Massimo Volpe, Maria Lorenza Muiesan, on Behalf of the SARS-RAS Investigators

There is an error in affiliation 17 for author Massimo Volpe. The correct affiliation should be two separate affiliations. The correct affiliation 17 is: Department of Clinical and Molecular Medicine, School of Medicine and Psychology, Sapienza University of Rome, Rome, Italy.

An affiliation for the 19^th^ author is not indicated. Massimo Volpe is affiliated with: IRCCS Neuromed, Sapienza University Sant’Andrea Hospital, Pozzilli (Isernia), Italy.
