# Correction: Gender differences in predictors of intensive care units admission among COVID-19 patients: The results of the SARS-RAS study of the Italian Society of Hypertension

**DOI:** 10.1371/journal.pone.0267622

**Published:** 2022-04-20

**Authors:** Guido Iaccarino, Guido Grassi, Claudio Borghi, Stefano Carugo, Francesco Fallo, Claudio Ferri, Cristina Giannattasio, Davide Grassi, Claudio Letizia, Costantino Mancusi, Pietro Minuz, Stefano Perlini, Giacomo Pucci, Damiano Rizzoni, Massimo Salvetti, Riccardo Sarzani, Leonardo Sechi, Franco Veglio, Massimo Volpe, Maria Lorenza Muiesan, on Behalf of the SARS-RAS Investigators

There is an error in the third sentence of the second paragraph of the Introduction section of this article [1]. Prior to the publication of this article [1], reference 6 was retracted by The New England Journal of Medicine and should not have been cited [2]. The correct reference is: WHO. Gender and COVID-19: Advocacy brief [Internet]. 2020 [cited 2021 Jun 10]. Available from: https://www.who.int/publications/i/item/WHO-2019-nCoV-Advocacy_brief-Gender-2020.1

As a result of this correction to the references, the following text in the second paragraph of the discussion is removed: “[odds ratio 0.79 (CI 0.65–0.95)] in an extensive observational database collecting patients from Asia, Europe, and the United States.”

The authors provide the following additional clarifications:

As stated in the article’s Discussion, the study design does not allow conclusions to be drawn about causal relationships. As such, the authors provide revised wording for the first sentence of the Conclusions section of the Abstract, and the first sentence of the Conclusions section of the Discussion. The correct sentences are, respectively, “Our study demonstrates that gender may be the primary determinant of the disease’s severity among COVID-19” and “Our study demonstrates a possible gender effect for women in COVID-19 that are protected from more severe clinical presentations of the disease.”

In the study registry [3], the registered timeframe for the secondary outcome “Number and type of anthropometric and clinical parameters that associate with COVID19 and COVID-19 severity” was 3 months. The article reports a shorter observation period between March 9th and April 29th 2020. The authors wish to clarify that the study design included a steering committee that performed an interim analysis. The number of required patients was collected more quickly than expected.
