# Supplementary material for: Gender differences in predictors of intensive care units admission among COVID-19 patients: The results of the SARS-RAS study of the Italian Society of Hypertension
Source: PLoS One. 2020 Oct 6;15(10):e0237297. doi: 10.1371/journal.pone.0237297 (PMC7537902; doi:10.1371/journal.pone.0237297)
Supplement: Attachment — Submitted filename: Rebuttal2.docx [file pone.0237297.s002.docx]

Reviewer #1.

Thank you.

Reviewer #2

Thank you for your revision. We have included the suggested changes and reworded throughout the manuscript the less fluent parts.
